# Response of phytoplankton to heavy cloud cover and turbidity in the northern Bay of Bengal

**DOI:** 10.1038/s41598-018-29586-1

**Published:** 2018-07-26

**Authors:** R. Jyothibabu, N. Arunpandi, L. Jagadeesan, C. Karnan, K. R. Lallu, P. N. Vinayachandran

**Affiliations:** 1 CSIR- National Institute of Oceanography, Regional Centre, Kochi, India; 2 Centre for Atmospheric and Ocean Sciences, Indian Institute of Science, Bangalore, India

## Abstract

An interesting physiological response of phytoplankton to large fluctuations in underwater photosynthetically active radiation (PAR) levels in the northern Bay of Bengal has been presented here. This study is primarily based on a 12-day time series observation in the northern Bay of Bengal during the peak Southwest Monsoon (July 2012), when the study region was recurrently exposed to alternating cloudy and sunny sky conditions. On overcast days, the PAR available underwater at the time series location (TSL) drastically decreased, with the noontime PAR at the surface water (2 m) usually being ~600 µmol m^−2^ s^−1^ on sunny days and declining to ~50 µmol m^−2^ s^−1^ on heavily overcast days. Closely linked with the sunny and cloudy days at TSL, chlorophyll *a* concentration in the water column showed noticeable features; it increased in the upper water column (surface-40 m) and decreased in the lower water column (41–80 m) on cloudy days, while the reverse was the case on sunny days. Based on *in-situ* and laboratory experimental data, it was observed that these temporal changes in the vertical distribution of chlorophyll *a* in the northern Bay of Bengal were due to the short-term physiological acclimation of phytoplankton to large changes in underwater PAR.

## Introduction

**Figure 1:**
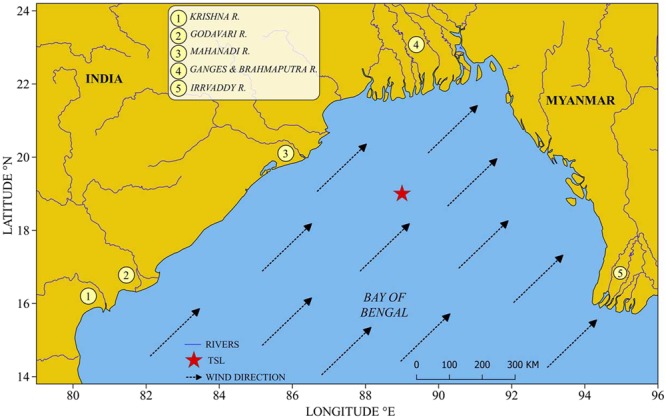
The location of TSL in the northern Bay of Bengal (red star). The dotted arrows heads indicate the wind direction during the Southwest Monsoon (July). Mouths of major Rivers are indicated by the numbers. This map was created using QGIS v2.18 (QGIS Development Team, 2017), following the attribution guidelines. QGIS Geographic Information System. Open Source Geospatial Foundation Project (http://www.qgis.org). QGIS software comes under GNU General Public License.

It has been recorded earlier in the Bay of Bengal that the vertical distribution of the high chlorophyll *a* layer in the water column varied zonally as well as seasonally. Murty *et al*.^1^ recorded an upward shoaling of the high chlorophyll *a* layer towards the coast in the western Bay of Bengal during the Southwest Monsoon, whereas, Gomes *et al*.^2^ and Jyothibabu *et al*.^3^ found such an upward vertical shift in the region during the Southwest Monsoon. Although there has been a general belief that phytoplankton stock can be affected by cloud cover and turbidity^2–7^, their influence on the phytoplankton physiology is unknown. This is particularly important as the cloud cover in this geographical area is rather transient (short-term), with heavily overcast as well as sunny days occurring alternately. It is also relevant to consider the fact that any oceanic area can experience heavily overcast days when atmospheric events such as depression and cyclones pass over that region. In this paper, we show that cloud cover and turbidity could induce a measurable short-term physiological response in the phytoplankton community inhabiting in the northern Bay of Bengal (Fig. 1). Major objectives of this paper were set (a) to understand whether cloud cover and turbidity could modify the vertical distribution of chlorophyll *a* in the northern Bay of Bengal as a physiological response of the phytoplankton community and (b) if so, what could be the possible mechanism that alters the vertical chlorophyll *a* distribution.

## Results

### Segregation of sampling days based on similarity

**Figure 2:**
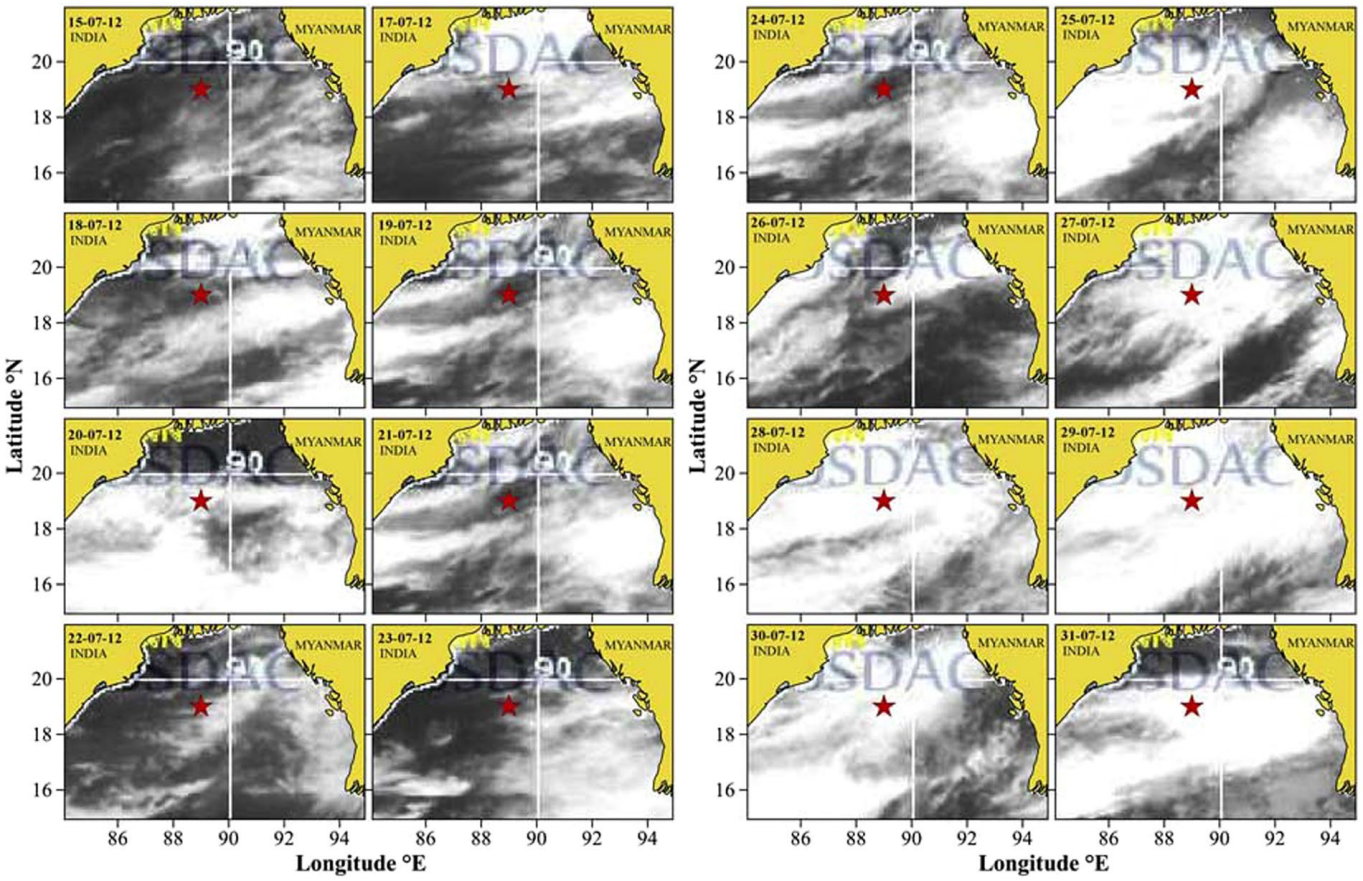
Cloud cover over TSL in the northern Bay of Bengal during the study period in July 2012 captured by INSAT KALPANA. The star mark indicate the location of TSL, which was sampled first on 15 July 2012. Later, daily time series measurements were carried out at TSL was from 20^th^ to 31^st^ July 2012. The white patches indicate cloud cover and dark patches clear sky conditions. The daily thermal infrared cloud imageries (Kalpana-1, https://www.mosdac.gov.in/) representing the time series observation period in the Bay of Bengal were downloaded and later digitalised and mapped using the tool QGIS (v2.18, QGIS Development Team, 2017) following the attribution guidelines (http://www.qgis.org). QGIS software comes under GNU General Public License.

**Figure 3:**
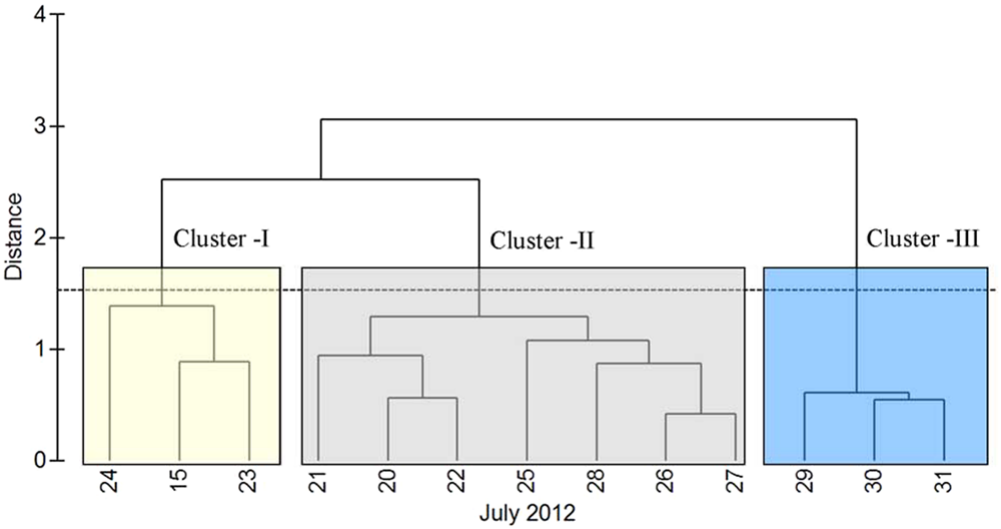
Euclidian distance matrix based cluster dendrogram showing grouping of sampling days based on their similarities in environmental parameters. Cluster I (CI) was characterised by relatively high salinity, high PAR and low turbidity, Cluster II (CII) with relatively high salinity, low PAR and low turbidity and cluster III (CIII) with relatively low salinity, low PAR and high turbidity. This analysis was done in PRIMER V6 (http://www.primer-e.com/).

During the time series, the TSL showed contrasting environmental settings as detailed in the Sampling and Methods section (CI, CII, and CIII). One of the most striking feature of the study region was the alternating periods of cloudy and sunny sky conditions (Fig. 2). In order to scientifically group the sampling days based on their similarity in environmental characteristics, Euclidian distance matrix-based clustering was used, the result of which is presented in Fig. 3. Three distinct environmental clusters were formed in the cluster/SIMPROF (P < 0.05) analysis in which cluster I was characterized by relatively high salinity, high PAR and low turbidity (Case I), cluster II with relatively high salinity, low PAR and low turbidity (Case II) and cluster III having relatively low salinity, low PAR and high turbidity (Case III). CI was formed of sampling days 15, 23 and 24, CII consisted of sampling days 20 to 22, and 25 to 28, and CIII consisted of days from 29 to 31 of July 2012. Hereafter in this paper, cluster I (CI) represents days with relatively high salinity, high PAR and low turbidity, cluster II (CII) consists of days with relatively high salinity, low PAR and low turbidity and cluster III(CIII) is of days with relatively low salinity, low PAR, and high turbidity.

### Cloud cover, wind, and rainfall

**Figure 4:**
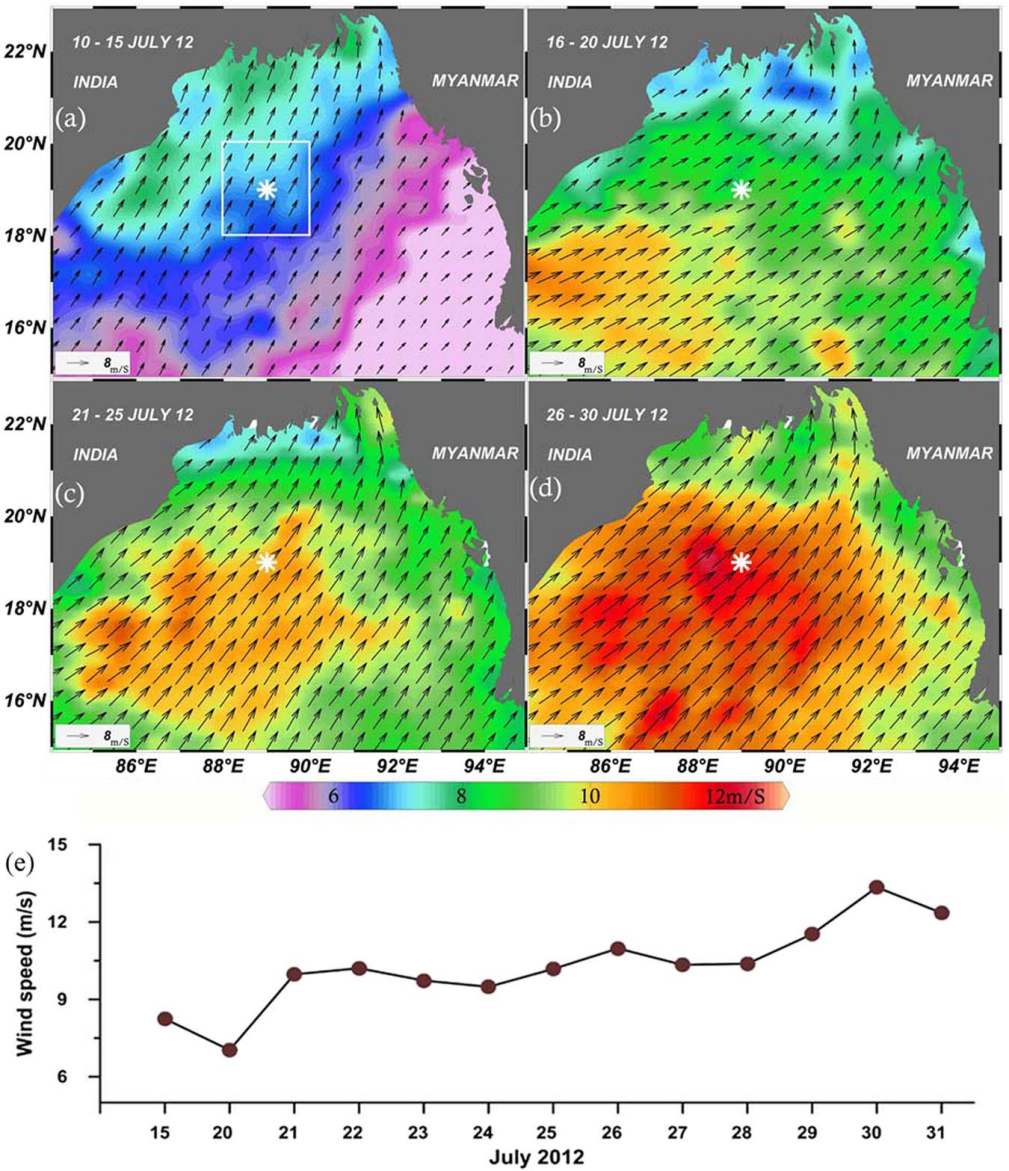
(**a**) Wind velocity over the field sampling at TSL. White asterisk symbol indicates the position of TSL. This spatial colour map represents the wind velocity and the direction. The direction of the arrows indicate the direction of the wind and the length of the arrows represent the wind speed in relation to the reference arrow. The magnitude of the wind speed in this spatial map is also represented in-terms of the colour scale. Wind speed Gradual increase of south-westerly winds at TSL with a peak during the last part of the sampling period is evident. (**b**) Daily mean wind over a 2° × 2° rectangle (Fig. 3a, panel 1) that encompasses TSL. The spatial map of the wind velocity was plotted using Ocean Data View following the attribution guidelines (Schlitzer, R., Ocean Data View, odv.awi.de, 2017). Ocean Data View software comes under GNU General Public License.

**Figure 5:**
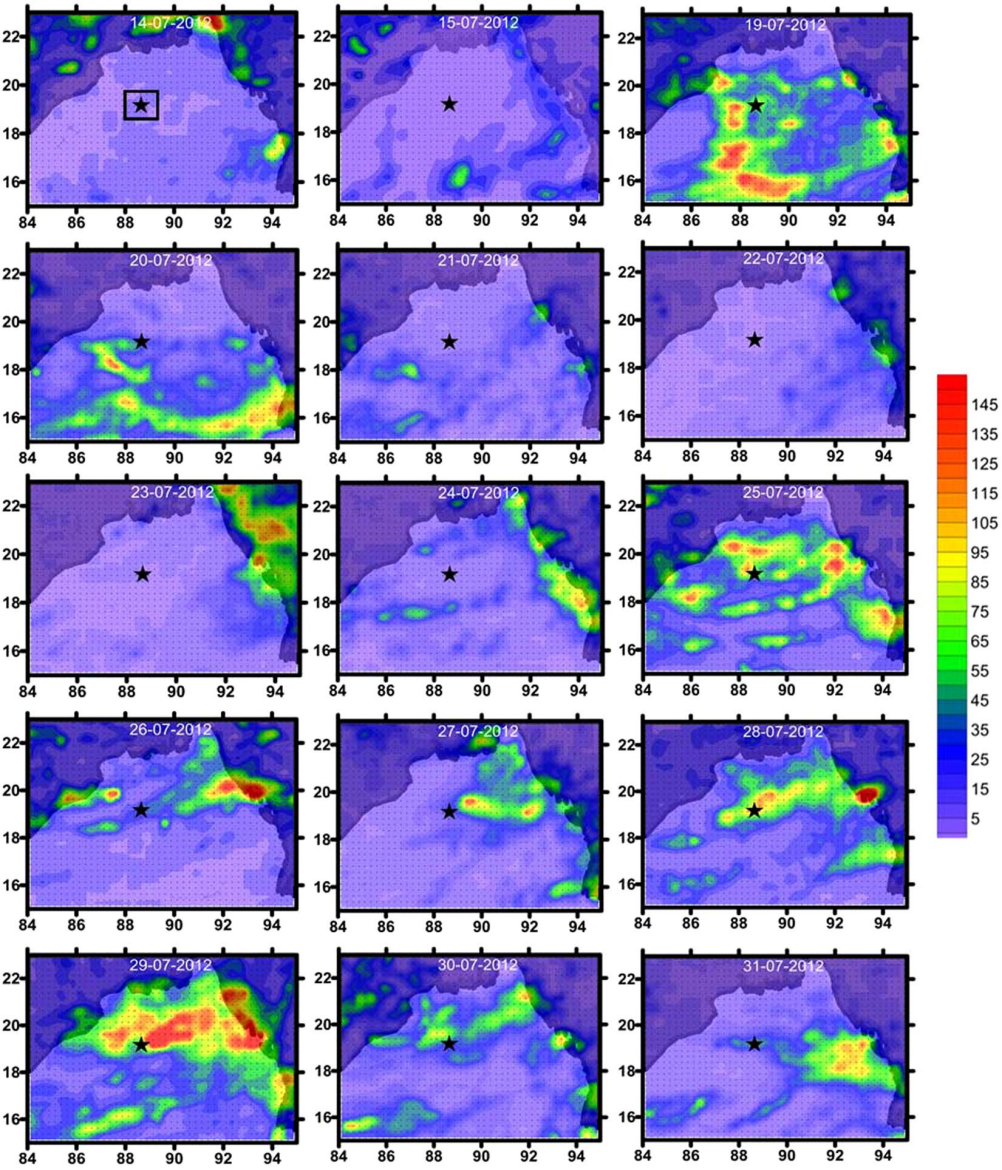
Daily rainfall (mm/day) over TSL (black star) during the sampling period. Heavy rainfall over TSL occurred on 19^th^, 25^th^ and 29^th^ July 2012. More importantly, increased rainfall in the catchment area of Mahanadi on 19^th^, 25^th^, 26^th^ and 29^th^ July are clear in this image. The rainfall maps were created using the Surfer software (version 9.1; Golden software, 2009) following the attribution guidelines (http://www.goldensoftware.com/).

The temporal change in cloud cover over the TSL is presented in Fig. 2. The time series of sky condition showed that cloud cover over the TSL was not a continuous feature during the sampling period. As mentioned above, the sampling days grouped as CI were all sunny, whereas CII and CIII were all heavily clouded days (Fig. 3). The temporal changes of surface wind in the study area (Fig. 4) showed a relatively weak south-westerly wind (6.8 m s^−1^) during CI, which then intensified and reached the peak (12 ms^−1^) during CII (Fig. 4a–d). A closer observation of the wind in the study area showed the peak during CIII (Fig. 4e). The rainfall in the study domain (Fig. 5) did not show a close linkage with the temporal variation in cloud cover (Fig. 2). Heavy rainfall was evident at the TSL on 19^th^ and 25^th^ and then on 28^th^ and 29^th^.

### Physico-chemical parameters

**Figure 6:**
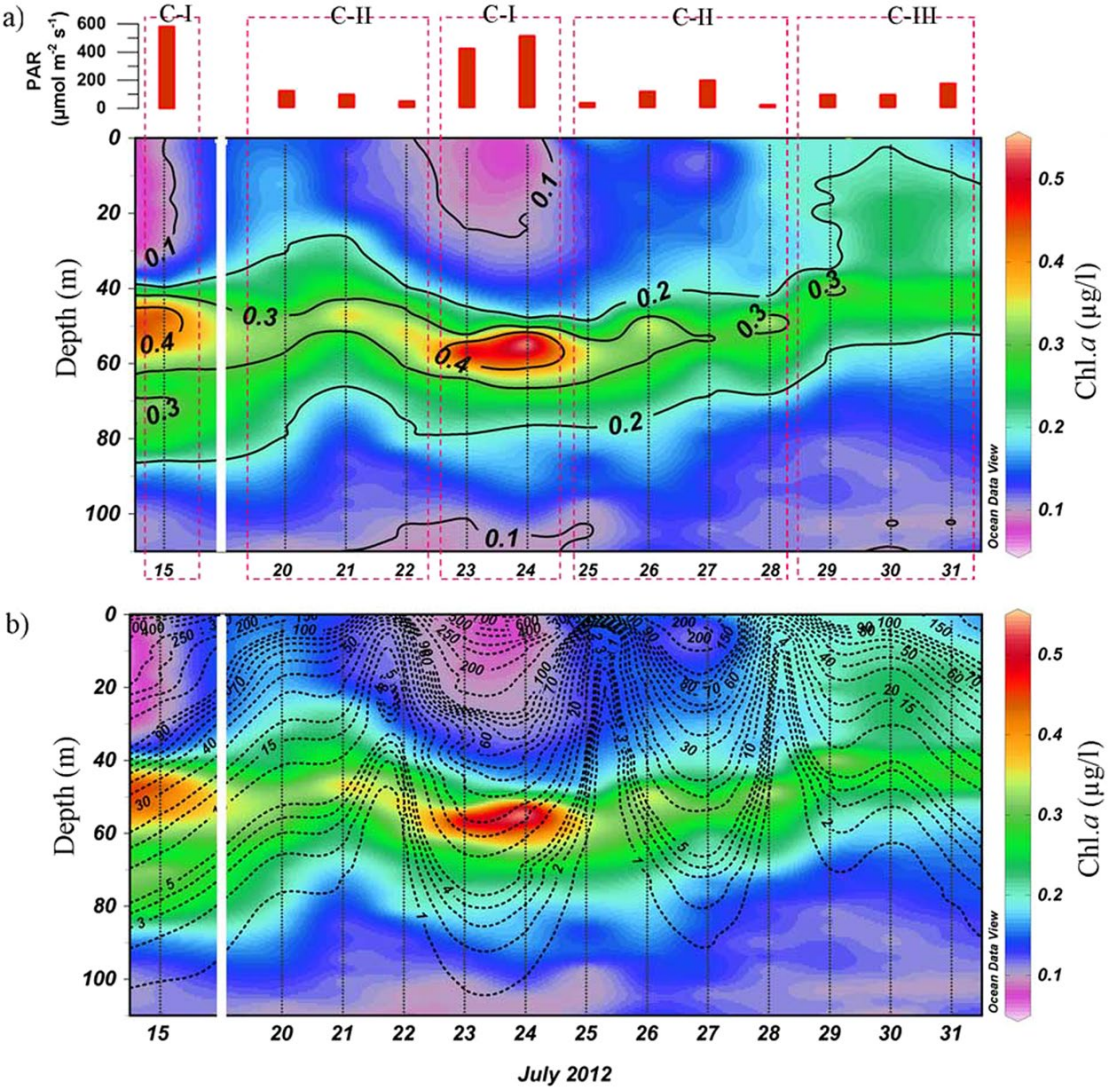
Temporal variations in the vertical distribution of chlorophyll *a* at TSL in the northern Bay of Bengal. Red coloured bars on the top panel indicate variation of PAR during the sunny and cloudy days. The contours in panel (**a**) represent chlorophyll *a* concentration while in panel (**b**) the PAR levels. Deepening and shallowing of the high chlorophyll *a* layer during the sunny and cloudy days are clearly represented in both panels. PAR was high on 15, 23 and 24 July (CI), which was associated with low concentration of chlorophyll *a* in the surface waters (0–40 m) and high concentration in the subsurface (41–80 m). On the other hand, 20, 21, 25–28 July with low PAR due to thick clouds (CII) and 28–31 with low PAR due to thick clouds and turbidity (CIII) increased the chlorophyll *a* in surface waters (0–40 m) and decreased in the subsurface (41–80 m) waters. The vertical distribution of the Chl.*a* (Contours plots) and PAR overlaid Chl.*a* plots (Contour plots) were plotted using Ocean Data View following the attribution guidelines (Schlitzer, R., Ocean Data View, odv.awi.de, 2017). Ocean Data View software comes under GNU General Public License.

The surface salinity was high at the TSL throughout the sampling period, which varied from 32.24–32.51 (Table 1). A decrease in salinity, though marginal, was observed during the environmental setting CIII. The mixed layer depth was moderate and varied from 30.7 to 31.6 m. The temporal variation of MLD showed a linkage with salinity drop with relatively low MLD during environmental setting CIII. The euphotic column, the water column above the depth at which PAR is 1% of the surface value, varied from 68–82 m and the lower values were observed on cloudy days having environmental setting CII and CIII. Turbidity varied from 0.2–0.8 NTU and showed the highest values during environmental setting CIII. During the time-series sampling period, PAR showed remarkable differences. During environmental setting CI, the sky was very clear with high surface PAR varying from 417–605 µmol m^−2^ s^−1^ and very low turbidity. During environmental setting CII, though PAR significantly decreased due to heavy cloud cover (21–180 µmol m^−2^ s^−1^), the turbidity and salinity showed only a marginal difference. The environmental setting CIII prevailed during the last three days of the time series, characterized by thick cloud cover, decrease in salinity and increase in turbidity; the surface PAR during this period varied from 30–80 µmol m^−2^ s^−1^ (Fig. 6). The nitrate concentration in the surface waters was low throughout the time series observations, which varied from 0.2 to 0.6 μM. Marginal increase in the concentration of nitrate was found during environmental setting CIII. Similar was the case of silicate, which was low (0.4–1.1 μM) throughout the observation period and increased marginally during environmental condition CIII.

**Table 1:** Environmental variables in three environmental settings CI, CII and CII.

	Parameters	C I	C II	C-III
1	Salinity	32.49 ± 0.12	32.48 ± 0.11	32.2 ± 0.02
2	Turbidity (NTU)	0.43 ± 0.03	0.54 ± 0.08	0.78 ± 0.04
3	Euphotic Depth (m)	72.3 ± 5.13	69.14 ± 1.95	66.33 ± 2.5
4	MLD (m)	31.3 ± 1.52	33. 28 ± 6.52	32.66 ± 3.2
5	Surface (2 m) PAR (µmol m^−2^ s^−1^)	502 ± 82	86 ± 61	116 ± 46
6	Nitrate (µM)	0.32 ± 0.03	0.39 ± 0.04	0.55 ± 0.01
7	Silicate (µM)	0.6 ± 0.17	0.7 ± 0.4	1.07 ± 0.1
8	Surface (2 m) chl. *a* (µg L^−1^)	0.09 ± 0.01	0.14 ± 0.02	0.19 ± 0.01
	0–80 m integrated chl. *a* (mg. Chl.*a*. m^−2^)	15.8 ± 1.3	15.6 ± 0.76	15.53 ± 0.4
	0–40 m integrated chl. *a* (mg. Chl.*a*. m^−2^)	4.16 ± 0.15	6.01 ± 0.79	8.13 ± 0.4
	41–80 m integrated chl. *a* (mg. Chl.*a*. m^−2^)	11.63 ± 1.3	9.57 ± 0.52	7.4
9	0–40 m phyt. abundance (cells L^−1^)	5540 ± 667	4970 ± 590	5940 ± 1380
	4–80 m phyt. abundance (cells L^−1^)	6200 ± 492	4940 ± 170	5100 ± 640
10	0–40 m chl. *a* per cell (pg cell^−1^)	18.9 ± 4.5	28.1 ± 6.5	30.7 ± 13.1
	41–80 m chl. *a* per cell (pg cell^−1^)	35.4 ± 9.7	58.6 ± 13.1	34.4 ± 9.3

Case I (CI) represents clear sky, high PAR, relatively high salinity and low turbidity, Case II (CII) stands for thick cloud cover/low PAR, relatively high salinity and low turbidity and Case III (CIII) corresponds to thick cloud cover/low PAR, relatively low salinity and high turbidity. MLD: Mixed Layer Depth; PAR: Photosynthetically Active Radiation).

### Vertical distribution of chlorophyll *a* and PAR

The vertical distribution of chlorophyll *a* in the water column during environmental settings CI to CIII at the TSL evidenced a few significant features (Table 1). First was the presence of a high chlorophyll *a* layer of almost 20–30 m thickness in the euphotic column. Second was the vertical shift of this high chlorophyll *a* layer on a temporal scale. Third was the close coincidence of the vertical shift of high chlorophyll *a* layer with the varying PAR underwater. The high PAR condition (CI) was characterized by very low surface chlorophyll *a* (<0.1 µg L^−1^) and prominently deep chlorophyll maxima (~0.6 µg L^−1^) at 50 m depth (Fig. 6). During low PAR and low turbidity condition (CII), chlorophyll *a* concentration doubled in the surface waters (0.1–0.2 µg L^−1^) and decreased to half in the deep chlorophyll maxima (0.3 µg L^−1^). Similar to the CII condition, there was a clear increase in chlorophyll *a* concentration in the surface and a decrease in sub-surface during CIII. More importantly, during CIII, the upward shift in high chlorophyll *a* layer was more prominent compared to CII (Fig. 6).

**Figure 7:**
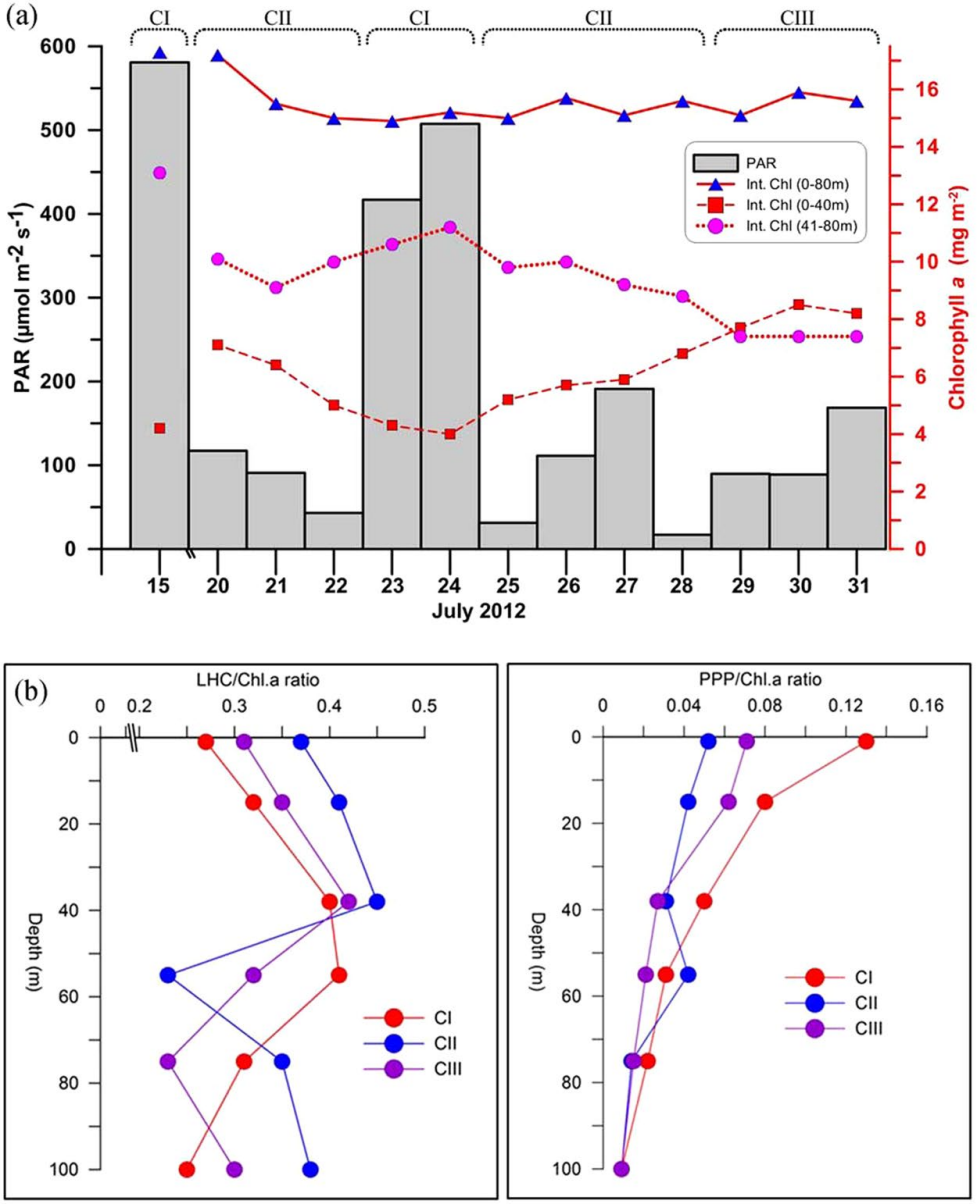
(**a**) Synchronous increase and decrease of integrated chlorophyll in the surface (0–40 m) and subsurface (41–80 m) layers during cloudy and sunny days. As these changes in chlorophyll *a* in the surface and subsurface layers were synchronous, the integrated chlorophyll *a* in the entire euphotic column (upper 80 m), represented in red line graph, remained without significant change. (**b**) Vertical distribution of Light Harvesting Pigments (LHP)/Chl. *a* and Photo Protective Pigments (PPP)/Chl.*a* in three different environmental conditions CI, CII and CIII. Plots are made in Grapher, V 7.2. (Golden Software, USA; http://www.goldensoftware.com/).

The vertical distribution of PAR was overlaid on chlorophyll *a* to show the correlation of these parameters (Fig. 6). It was evident that when surface PAR was >200 µmol m^−2^ s^−1^ (CI), the chlorophyll *a* concentration noticeably decreased in the upper euphotic column (upper 40 m) and increased in the lower euphotic column (41–80 m). Reverse was the situation when surface PAR was <200 µmol m^−2^ s^−1^ while environmental setting CII and CII prevailed. It was also clear that chlorophyll *a* maxima in the water column was found where PAR was <50 µmol m^−2^ s^−1^. In order to understand the temporal shift in the vertical distribution of high chlorophyll *a* layer in the water column more clearly, integrated chlorophyll *a* concentration in the upper (0–40 m) and lower (41–80 m) water column was analysed and the results showed several noticeable features (Fig. 7).

Firstly, the total chlorophyll *a* content in the upper water column (upper 40 m) was much lower than in the lower water column (41–80 m). Secondly, closely linked to the recurring sunny (CI) and cloudy days (CII and CIII), there was a synchronous but reverse change in the chlorophyll *a* concentration in the upper (upper 40 m) and lower water column (41–80 m). On cloudy days (CII and CIII), chlorophyll *a* concentration in the upper water column increased while it decreased in the lower water column; the reverse was the case on sunny days (Fig. 7a). Thirdly, as the increase and decrease of chlorophyll *a* in the surface and subsurface waters were synchronous and reverse, the integrated chlorophyll *a* of the entire water column (upper 80 m) remained roughly the same throughout the time series. In other words, the integrated chlorophyll *a* concentration in the upper 80 m water column was more or less same irrespective of the short-term vertical variations in chlorophyll *a* concentration during the sunny and cloudy days.

### Distribution of light harvesting and photoprotective pigments in relation to PAR

The vertical distribution of light harvesting pigments (LHP) and photoprotective pigments (PPP) in phytoplankton cells in the water column and their temporal variations with respect to different light conditions (CI, CII, and CIII) are presented in Fig. 7b. LHP was found to be lower in phytoplankton throughout the water column under CI condition compared to CII and CIII. Vertically, LHP concentration in phytoplankton under CII and CIII conditions peaked at 40 m depth and then gradually decreased downwards. Although such a clear vertical peak was not found, LHP under CI condition also showed a gradual increase from the surface up to 60 m and then a decrease downwards. In the case of PPP, a clear increase in concentration was observed during CI condition as compared to CII and CIII conditions. Overall, from the surface to the deeper waters in the study region, there was a general decrease in concentration of PPP in phytoplankton cells.

### Phytoplankton cell abundance and chlorophyll *a* per cell

**Figure 8:**
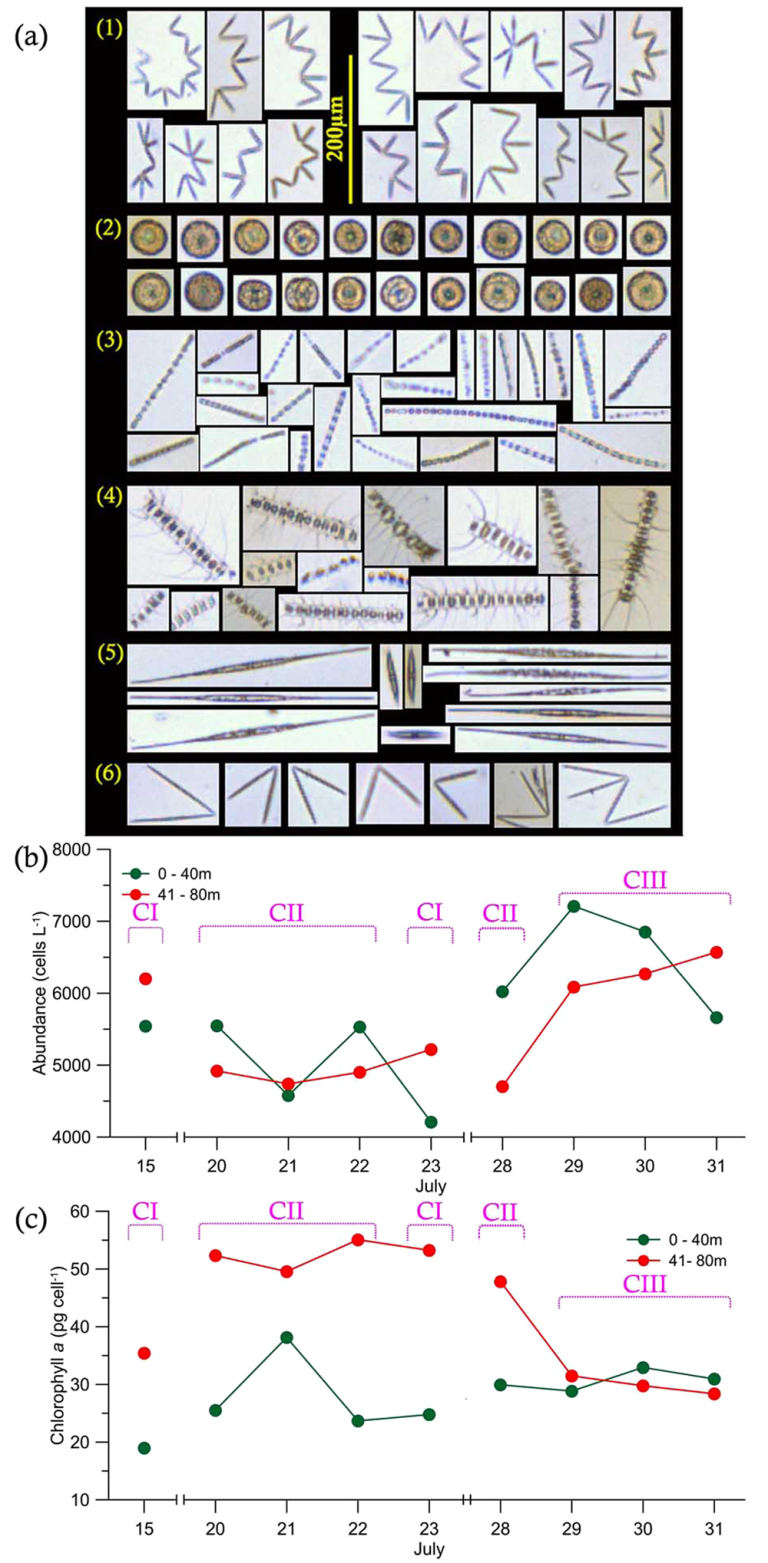
(**a**) FlowCAM images showing the dominant phytoplankton (1) *Thalassionema* (2) *Thalassiosira* (3) *Skeletonema* (4) *Chaetoceros* (5) *Nitzschia* and (6) *Thalassiothrix* and phytoplankton mean (**b**) abundance and (**c**) chlorophyll *a* content per phytoplankton in the upper and lower euphotic column at TSL during the observations. Unlike in Figs 6 and 7a, there was no data in this Figure for 25–28^th^ July, which is indicated by additional splits on the x axes.

The phytoplankton community at the TSL consisted mostly of diatoms and relatively fewer dinoflagellates (Fig. 8a). Diatoms such as *Skeletonema*, *Thalassiothrix, Thalassionema, Chaetoceros*, *Thalassiosira Nitzschia*, and *Coscinodiscus* dominated the diatom community at the TSL throughout the observations. The dinoflagellate component consisted of *Ceratium*, *Podolamphas*, and *Heterolaucus*. The variations in cell abundance of phytoplankton in the water column are presented in Fig. 8b, which neither shows any clear temporal pattern nor any noticeable linkage with the observed temporal pattern in chlorophyll *a* vertical distribution. The temporal variation in the chlorophyll *a* content per phytoplankton cell in the upper water column (0–40 m) is presented in Fig. 8c, which shows an increase in chlorophyll *a* in phytoplankton cells in the upper water column when environmental conditions CII and CIII exist. Similarly, there was an increase in chlorophyll *a* concentration in phytoplankton cells in the lower water column (41–80 m) when high surface PAR was available, as during environmental setting CI. This pattern was very similar to the spatial variations of chlorophyll *a* concentration observed in the surface and subsurface waters during sunny and cloudy days presented in Figs 6 and 7a.

### Interrelationships of parameters from RDA

**Figure 9:**
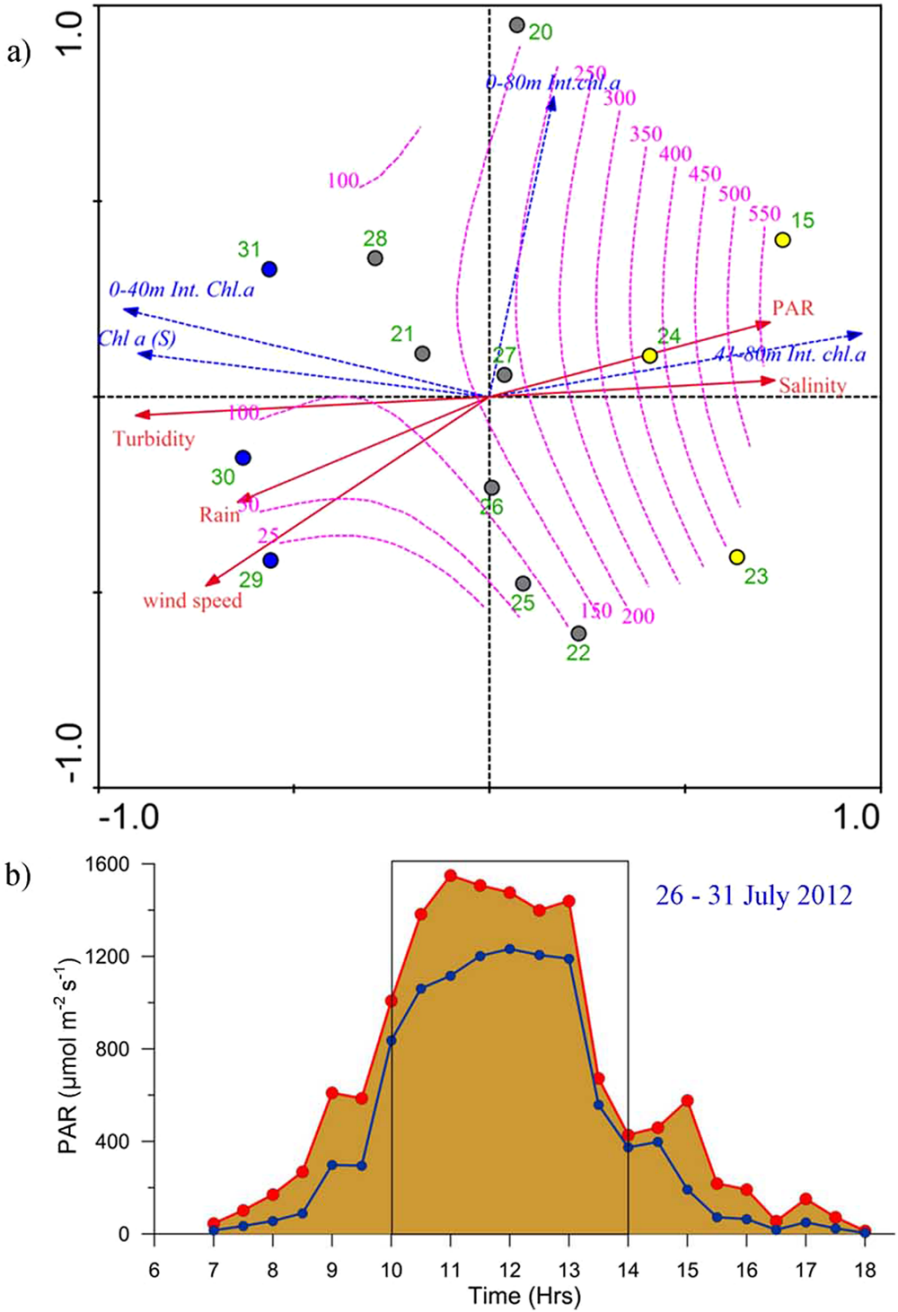
(**a**) The ecological inter-relationships evidenced in RDA analysis. Filled dotted circles indicate sampling days. Yellow filled circle represent environmental setting CI, black circles CII and blue CIII. The PAR level during the sampling period was overlaid as purple dotted lines over other parameters and (**b**) temporal variations in Photosynthetically Active Radiation (PAR) during the experimental period (10 to 14 hrs.). PAR in surface water was indicated by red line and PAR at 0.5 m in blue line. PAR measurements were carried out every half an hour. Plot (**a**) are made in by CANOCO 4.5 plot using the standard procedure of the Leps, J. & Smilauer, P.S^43^ (http://www.microcomputerpower.com/default.html) (**b**) using Grapher, V 7.2. (Golden Software, USA; http://www.goldensoftware.com/).

Result of RDA analysis depicting the inter-relationships of environmental parameters has been presented in Fig. 9, which provides conclusive features. The environmental variables explained 89.3% of the chlorophyll *a* distribution. The ordination significance of all the axes was tested by a Monte Carlo procedure, which showed that all the ordinations of RDA axes were significant (F ratio − 11.69 and P < 0.005). In RDA Triplot, samples are represented as points, and quantitative environmental and biological variables as arrows. The direction of an arrow indicates the direction of increase of that variable and the angle between two arrows represent the significance of correlation. It was evident through RDA that the chlorophyll *a* concentration in the surface waters as well as upper water column (0–40 m) was positively correlated with high turbidity and negatively correlated with underwater PAR. Similarly, the chlorophyll *a* concentration in the lower water column (41–80 m) positively correlated with underwater PAR and high salinity and negatively correlated with turbidity. It was also observed that wind, rainfall, and turbidity were closely linked during the observation period. Also, positively correlated was the turbidity and wind while indicating the environmental setting CIII. The overlaid contours of PAR showed that the surface and integrated chlorophyll *a* in the upper water column (0–40 m) was less in cases where PAR was >200 µmol m^−2^s^-1^ (CII and CIII).

### Salient observations from experiments

The results of the experiments (Table 2) showed that the phytoplankton cell abundance in the experimental bottles before and after the incubation experiments were comparable and, therefore, their temporal variation was statistically insignificant (P > 0.05). On the other hand, the total chlorophyll *a* concentration before and after the incubation showed large and statistically significant variations (P < 0.01). PAR fluctuation in the experimental bottles during the incubation period as part of the natural daily cycle is presented in Fig. 9b. Over the experimental incubation period, chlorophyll *a* in the experimental bottles exposed to high light condition (400–1200 µmol m^−2^ s^−1^) noticeably decreased (a decrease of 14% in *Skeletonema*; 12% in *Chaetoceros*; 8% in *Thalassiosira*) from the control bottles. On the other hand, after the experimental incubation of the phytoplankton exposed to low light condition (40–120 µmol m^−2^ s^−1^), the chlorophyll *a* concentration noticeably increased (an increase of 7% in *Skeletonema*; 21% in *Chaetoceros* and 49% in *Thalassiosira*) from the control values. This trend was clearer in the case of experimental bottles exposed to the lowest light condition (PAR 4–12 µmol m^−2^ s^−1^), wherein an increase in chlorophyll *a* concentration was observed after the incubation period (an increase of 24% in *Skeletonema*; 30% in *Chaetoceros* and 75% in *Thalassiosira*) as compared to the control bottle values.

**Table 2:** Results of the incubation experiments.

Parameters	Control (150–300 µmol m^−2^ s^−1^)	High (400–1200 µmol m^−2^ s^−1^)	Low (40–120 µmol m^−2^ s^−1^)	Lowest (4–12 µmol m^−2^ s^−1^)
***Skeletonema***				
Abundance (×10^6^) Cells L^−1^	3.46 ± 0.03	3.29 ± 0.08	3.53 ± 0.06	3.81 ± 0.05
Per cell Chl.*a* (Pg.Chl.*a*)	6.91 ± 0.04	6.16 ± 0.06	8.53 ± 0.08	9.58 ± 0.09
LHP/Chl.*a* ratio	0.436 ± 0.014	0.396 ± 0.09	0.512 ± 0.018	0.817 ± 0.066
PPP/Chl.*a* ratio	0.044 ± 0.002	0.136 ± 0.001	0.048 ± 0.006	0.027 ± 0.004
***Chaetoceros***				
Abundance (×10^6^) Cells L^−1^	24.46 ± 0.08	24.21 ± 0.02	25.31 ± 0.04	25.53 ± 0.08
Per cell Chl.a (Pg.Chl. *a*)	0.54 ± 0.03	0.49 ± 0.01	0.63 ± 0.06	0.70 ± 0.08
LHP/Chl.*a* ratio	0.377 ± 0.016	0.277 ± 0.04	0.463 ± 0.013	0.535 ± 0.01
PPP/Chl.*a* ratio	0.046 ± 0.001	0.092 ± 0.003	0.049 ± 0.004	0.021 ± 0.002
***Thalassiosira***				
Abundance (×10^6^) Cells L^−1^	24.49 ± 0.13	24.41 ± 0.08	24.81 ± 0.11	25.35 ± 0.12
Per cell Chl.*a* (Pg.Chl.*a*)	0.21 ± 0.09	0.20 ± 0.06	0.31 ± 0.04	0.39 ± 0.06
LHP/Chl.*a* ratio	0.308 ± 0.005	0.237 ± 0.019	0.379 ± 0.015	0.978 ± 0.076
PPP/Chl.*a* ratio	0.042 ± 0.005	0.070 ± 0.001	0.046 ± 0.003	0.036 ± 0.003

LHP - Light Harvesting Pigments and PPP - Photo Protective Pigments. In relation to PAR available during the experimental period, 4 intensities were maintained in the experimental bottles - 100% (high), 10% (low) and 1% (lowest). ‘Control’ was meant to simulate the PAR range found in the field when the experimental sample was collected. Also the same PAR level was maintained in the environmental chamber while developing the cultures of diatoms for the experiments. The experimental bottles were incubated for 4 hours during the peak daylight hours (10 to 14 hours).

The result of the phytoplankton per cell chlorophyll *a* before and after the incubation experiments is presented in Table 2. It is clear that the chlorophyll *a* content per cell of the phytoplankton decreased in experimental bottles with high light conditions (400–1200 µmol m^−2^ s^−1^) as compared to the control bottles. On the other hand, per cell chlorophyll *a* in phytoplankton noticeably increased in the experimental bottles exposed to the low (40–120 µmol m^−2^ s^−1^) and the lowest (4–12 µmol m^−2^ s^−1^) light conditions as compared to the control bottles. The overall results showed that the cell abundance of phytoplankton did not change much during shorter timescales, whereas per cell concentration of chlorophyll *a* in phytoplankton cells varied over shorter time scales in response to large variations in PAR. Six photosynthetic pigments measured from the experimental samples using HPLC, namely, fucoxanthin, alloxanthin, diatoxanthin, diadinoxanthin, β carotene and chlorophyll *a* were used to interpret the experimental data. Out of these, fucoxanthin is an LHP, whereas diatoxanthin, diadinoxanthin, alloxanthin and β carotene are PPP. At the end of the experiments, the ratio of LHP/Chl.*a* and PPP/Chl.*a* in the incubation bottles showed some significant features. The ratio of LHP/Chl.*a* was highest in the bottles exposed to the lowest PAR level (4–12 µmol m^−2^ s^−1^), followed by the bottles exposed to low PAR level (40–120 µmol m^−2^ s^−1^), being the least in the bottles exposed to the high PAR level (400–1200 µmol m^−2^ s^−1^). On the other hand, the ratio of PPP/Chl. *a* showed the highest values in the bottles exposed to the highest PAR level (400–1200 µmol m^−2^ s^−1^) followed by the bottles exposed to low PAR level (4–12 µmol m^−2^ s^−1^); the value was least in the bottles exposed to the lowest PAR level (40–120 µmol m^−2^ s^−1^).

## Discussion

The most important features recorded in the present study were the presence of a high chlorophyll *a* layer of almost 20–30 m thickness in the euphotic column, and their vertical shift on a temporal scale coinciding with varying PAR underwater. The study showed that chlorophyll *a* maxima at the TSL occurred at depths where PAR was significantly low (<50 μmol m^−2^ s^−1^). This corroborated with the earlier observation from the Bay of Bengal that low irradiance levels persist in the region of the subsurface chlorophyll *a* maxima, suggesting that phytoplankton within the subsurface chlorophyll *a* maximum were light-limited^2^. It was also observed in the present study that on cloudy days (CII and CIII), chlorophyll *a* concentration increased and decreased in the upper euphotic column (upper 40 m) and lower euphotic column (41–80 m), respectively. To analyse this feature more clearly, it was ideal to follow the view of Kiefer^8,9^, who showed that there are two causes of variability in chlorophyll concentration in the sea: those caused by changes in the concentration of particles that contain chlorophyll *a*, and those caused by the changes in the concentration of the chlorophyll *a* within these particles.

The analysis of phytoplankton abundance data showed that short-term variations in the numerical abundance of phytoplankton cells in the surface and subsurface waters at the TSL were not able to explain the observed temporal pattern in the vertical distribution of chlorophyll *a*. On the other hand, the temporal changes in chlorophyll *a* content per phytoplankton cell evidenced a close positive linkage with the observed change in the vertical distribution of chlorophyll *a* in the surface and subsurface waters. Therefore, it is inferred here that the synchronous changes in chlorophyll *a* in the surface and subsurface waters observed at the TSL were due to the short-term physiological adaptation of phytoplankton cells through photo-acclimation and chlorophyll reorganization in response to the large changes in PAR levels. The present study is a first-of-its-kind attempt from the field, showing the natural phytoplankton community response to large fluctuations in underwater PAR levels.

The ecological interrelationships in RDA analysis presented how turbidity influenced the vertical distribution of chlorophyll *a* at the TSL. The net effect of increased turbidity was the decreased PAR underwater, and its influence on phytoplankton short-term response was clearly represented during environmental setting CIII. The mean PAR level at the surface water during CIII was found to be higher than CII, but increased turbidity during the former period caused a noticeable increase in chlorophyll *a* concentration in the upper euphotic column. Also, in such cases (CIII), the usually well-marked subsurface chlorophyll *a* maxima was absent. In fact, the presence of high chlorophyll *a* concentration in the upper euphotic column without clear deep chlorophyll maxima has been noticed earlier as a typical feature of the coastal Bay of Bengal during the Southwest Monsoon as a result of high turbidity and cloud cover^2^. The rainfall data presented in Fig. 5 shows noticeably higher rainfall over Mahanadi catchment area a few days before (25^th^ and 26^th^ July) the CIII conditions at the TSL (29^th^ to 31^st^ July).

Globally, subsurface chlorophyll *a* maximum is a general characteristic of the oligotrophic ocean euphotic layer^2,10–14^. In the past, several hypotheses were put forward to explain the existence of deep chlorophyll *a* maximum^10,11,14,15^ and most of these were disproved in later studies^14,16^. The hypothesis that remained less criticized argues that the subsurface chlorophyll *a* maximum is a physiological adaptation (photo-acclimation) of phytoplankton cells to low light conditions^17–19^. It has been noticed in several studies that marine phytoplankton acclimate to rapid changes in light conditions and oligotrophic waters, and the vertical chlorophyll maximum does not represent a biomass maximum of phytoplankton but an increased chlorophyll per biomass at low light levels^20–22^. The phytoplankton community in the surface waters of tropical oceans grows under high light conditions with low chlorophyll *a* content^18,21^. It was also noticed that chlorophyll *a* per phytoplankton increases with depth above the chlorophyll *a* maximum where phytoplankton adapts to decreasing PAR by increasing its capacity to absorb light. Maxima in phytoplankton biomass occur where the growth rate is balanced by losses (respiration and grazing) and the divergence in sinking velocity, whereas the vertical distribution of chlorophyll is strongly determined by photo-acclimation.

Also, it is important to recall here that most of the phytoplankton usually has a time lag in responding to the nutrient enrichment in the environment and increase in their cell counts/chlorophyll biomass, which may vary from a few hours to a few days depending upon the species present in the environment^23^. Therefore, short-term variations in the vertical distribution of chlorophyll *a* observed in the northern Bay of Bengal are of little relevance when linked with any water column dynamics induced by wind or other disturbances. Also, the mixed layer and nitrate concentration in the surface waters did not show any significant variations during different phases of the time series observations. Therefore, it has been proposed here that the observed fluctuations in the vertical distribution of chlorophyll *a* observed in the northern Bay of Bengal was not TSL-specific, but was a general response of the entire northern Bay of Bengal to the large variations in underwater PAR levels. This argument was supported by the incubation experiments conducted in the present study, which clearly showed that the phytoplankton cells exposed to high/lowest solar radiation, even for shorter time scale, can change the ratio of the PPP/LHP as an adaptive mechanism to cope with high or low light conditions. This process was the basic reason for the observed vertical fluctuation in the positioning of high chlorophyll *a* layer at the TSL when there were large fluctuations in underwater PAR due to heavy cloud cover and turbidity. It is fundamental to consider PAR as the most important environmental variable governing the primary production and photosynthetic efficiency in phytoplankton^24,25^. Under low light conditions, most of the phytoplankton carry out biochemical reorganization of the intracellular photosynthetic mechanism to effectively collect even the scarce photon flux available by adjusting the ratio of the LHP to the reaction center^24–26^. On the other hand, phytoplankton cells exposed to excess light conditions, as in the case of the surface phytoplankton in the tropical oceans, increase the relative amount of PPP in their photosynthetic mechanism in order to protect the photosynthetic unit from photo-damage due to excess solar radiation. In diatoms, fucoxanthin is the major LHP, whereas xanthophylls [diatoxanthin (DD) and diadinoxanthin (DT)] are the PPP. In phytoplankton cells, the conversion between xanthophylls occurs very rapidly (seconds to minutes); therefore, on a shorter time scale, the sum of DD and DT remains the same^24–26^. The ratios of chlorophyll *a* (Chl. *a*)-specific xanthophyll pool [PPP (DD + DT)/Chl. *a*] and the ratios of chlorophyll *a* (Chl. *a*)-specific light-harvesting pigment (LHP/Chl.*a*) are generally used as indices of photoprotection and photoadaptation, respectively^27–29^. The observations mentioned above are strongly supported by one of the recent laboratory culture experiments of phytoplankton from the northern Bay of Bengal, which clearly showed that chlorophyll *a* concentration noticeably increases under decreased light condition compared to 100% saturated light^30^.

Earlier studies in the Bay of Bengal detailing the vertical distribution of chlorophyll *a* provide conflicting observations, but most of them can be explained logically based on the present study. Photo-inhibition is a typical feature of the ocean surface in tropical oceans, and taking this feature and increased cloud cover in the Bay of Bengal into consideration, Qasim^31^ supposed that the cloud cover over the Bay of Bengal might cause the lack of photo-inhibition at the surface waters. Later, Gomes *et al*.^2^ and Jyothibabu *et al*.^3^ showed that high chlorophyll *a* concentration and productivity maxima occurred in the surface waters of the Bay of Bengal only during the Southwest Monsoon when underwater light levels were significantly low. They also showed that during the cloudy Southwest Monsoon period, the high chlorophyll *a* concentration in the surface waters does not match with high productivity values. In the light of the present study, it appears that the supposition of Qasim^31^ and the observations of Gomes *et al*.^2^ and Jyothibabu *et al*.^3^ were logically correct, although there is a certain level of uncertainty in the former case wherein the lack of photo-inhibition was suggested as an annual feature of the Bay of Bengal, which is not true^2,3^. Studies of Murty *et al*.^1^ and Sarma and Aswanikumar^32^ showed a clear deepening of the high chlorophyll layer towards the offshore and presented a general trend wherein clear deep chlorophyll *a* maxima appeared at 30–50 m in the coastal region and 50–100 m in the open sea in the Bay of Bengal. Considering the significant decrease in turbidity and light attenuation towards offshore in the Bay of Bengal, the above observations on the orientation of the high chlorophyll *a* layer in costal-offshore transects are explainable. Obviously, the coastal waters of the Bay of Bengal have high light attenuation compared to the offshore waters^33^, and this might eventually lead to the upward shift of chlorophyll maximum layer in the former region.

## Conclusion

The short-term impact of cloud cover and turbidity on the vertical distribution of chlorophyll *a* in the northern Bay of Bengal has been presented here. Based on laboratory experiments supported with HPLC photosynthetic pigment analysis, it has been shown here that the temporal changes in the vertical distribution of chlorophyll *a* observed in the northern Bay of Bengal during the Peak Southwest Monsoon was due to the short-term physiological adaptation of phytoplankton cells through photoacclimation and chlorophyll re-organization to adapt with large changes in PAR. The present study also attempted to explain some of the earlier contrasting observations on the vertical orientation of the high chlorophyll *a* layer in the Bay of Bengal, pointing out the relevance of sky conditions and light attenuation of the water column in such cases.

## Sampling and Methods

This study was conducted in the open ocean region of the northern Bay of Bengal as a part of the Ministry of Earth Sciences (Govt.of India) funded project and therefore prior permission from any authorities for conducting oceanographic surveys in the region was not required. Also, the present study was aimed to understand the biophysical linkages of plankton in the study area, and the experimental organisms (phytoplankton) do not fall under any endangered/ protected category in this part of the world. Oceanographic datasets collected from a Time Series Location (TSL) in the northern Bay of Bengal (Lat. 19°N; Lon. 89°E) during the peak (15–31^st^ July) Southwest Monsoon (Fig. 1) are used in this study. The TSL was situated reasonably far from the landmasses and immediate reach of freshwater influx from rivers (Fig. 1). However, it has been recorded that when strong winds prevail over the study domain, the TSL is influenced by the low saline waters from the Indian coast^34^. Under this unique environmental setting at the TSL, we recorded the response of phytoplankton community to three typical environmental settings of the northern Bay of Bengal: Case I (CI) - clear sky/ high PAR, relatively high salinity and low turbidity, Case II (CII) - thick cloud cover/low PAR, relatively high salinity and low turbidity and Case III (CIII) - thick cloud cover/low PAR, relatively low salinity and high turbidity. During field sampling at the TSL in July 2012, a rare opportunity was obtained to sample all the three environmental cases and to record the short-term physiological response of phytoplankton to such environmental cases. The field sampling at the TSL was carried out in July 2012 on-board *ORV Sagar Kanya* as a part of the Continental Tropical Convergent Zone (CTCZ) programme of the Ministry of Earth Sciences, Govt. of India. The TSL was occupied first on 15^th^ July 2012, when the sky was clear, which enabled us to record the phytoplankton response to environmental setting I. From July 19^th^ onwards, the TSL was occasionally covered by thick monsoon clouds (Fig. 2) and we re-occupied the TSL on 20^th^ July 2012 for initiating the daily time series measurements for the next 12 days (till 31^st^ July 2012), during which the phytoplankton response to environmental settings II and III were measured. All oceanographic samplings during the time series were carried out during noon time. In addition to the collection of standard oceanographic datasets from the TSL, relevant satellite remote sensing data were also used in this study to make the field observations more robust and conclusive.

A Seabird Conductivity Temperature Depth (CTD) profiler fitted with additional sensors for Photosynthetically Active Radiation (PAR) and chlorophyll *a* were used for recording the vertical profiles of the respective parameters. From the CTD density data, the mixed layer depth (MLD) was calculated as the depth at which the density value increases by 0.2 kg m^−3^ than the surface value. The wet lab eco-fluorometer sensor attached to the CTD rosette was used to record the chlorophyll *a* (every 1 m) from the water column (avoid repetition). PAR underwater was measured using a PAR sensor, which was used to determine the euphotic depth. A turbidity meter (Eutech) was used to measure the turbidity of the surface water. Nutrients (nitrate and silicate) were measured using an auto-analyser (SKALAR) following standard colorimetric principles^35^. Water samples for nutrients and chlorophyll were collected from standard depths (0, 10, 20, 30, 40, 60, 80 and 100 m) using Niskin samplers attached to the CTD rosette. The phytoplankton samples from standard depths were filtered through GF/F Filters and measured using a lab Turner fluorometer (Trilogy, Turner designs, USA) following standard procedures of UNESCO^36^. Phytoplankton samples collected from selected depths (0, 20, 40, 60, 80 and 100) on days representing CI, CII, and CIII were filtered through GF/F filters and the samples were analysed for various phytoplankton photosynthetic pigments using High-Performance Liquid Chromatography (HPLC) following standard protocols (van Heukelem, 2002). The laboratory fluorometer data of chlorophyll *a* was used to standardize the CTD fluorometer data^1,18,37,38^ and the regression analysis between the two datasets showed a strong positive relation (y = 1.057 × + 0.070; R² = 0.889; Measured Chl. a = 1.057* CTD Chl. *a* + 0.070 R^2^ = 0.889; Supplementary Fig. S1). The standardized CTD fluorometer data was used to generate high-resolution vertical distribution plots of chlorophyll *a* at the TSL.

Water samples (1 liter) were collected at standard depths for determining phytoplankton diversity and abundance. These samples were analysed by a FlowCAM (Fluid Imaging Technologies, USA) following standard procedures^39–42^. The abundance of phytoplankton and total chlorophyll *a* data were used to calculate chlorophyll *a* per phytoplankton cell. The multivariate statistical method Redundancy Analysis (CANOCO 4.5) was used to ascertain the linear relationships among physicochemical and biological variables. The ordination significance was tested with Monte Carlo permutation tests (499 unrestricted permutations) (p < 0.05). The result of RDA analysis has been presented as Triplots in which samples are represented as points, and quantitative environmental and biological variables as arrows. The direction of an arrow in a Triplot indicates the direction of increase of that variable and the angle between two arrows represents how significantly they are correlated^43^. Based on the Triplot, the inter-relationships between various environmental and biological parameters, in terms of their statistical significance, are presented as a correlation matrix^43^. Considering salinity, PAR and turbidity as major environmental parameters influencing the vertical phytoplankton distribution at the TSL, Euclidian distance matrix-based cluster/SIMPROF analysis were carried out using Primer V6 to show the grouping of sampling days^44^.

### Laboratory experiments

In order to interpret the field data of chlorophyll *a* more precisely with respect to underwater PAR, laboratory experiments were carried out using dominant diatoms inhabiting the north-western Bay of Bengal. Initially, 80 liter of natural seawater collected from North-western Bay of Bengal was gently filtered through a 60 µm nylon mesh to remove the dominant plankton grazers (copepods and microzooplankton). The grazer-free phytoplankton community in the filtrate was concentrated gently on a 5 µm bolting silk and then transferred into clean glass bottles enriched with F/2 culture medium. The mixed phytoplankton culture thus developed was composed of *Skeletonema* sp., *Chaetoceros* sp. and *Thalassiosira* sp., which are the dominant diatoms in the North-western Bay of Bengal. These diatoms were isolated through serial dilution and then developed into pure cultures using F/2 medium in an environmental chamber (Sanyo, Model MLR-351H). Subsequently, subcultures were developed once in every 5 days, which were maintained under 12:12 hrs light and dark cycle with a day time PAR 150–300 µmol m^−2^ s^−1^. On the 3^rd^ day of the 2^nd^ set of the subculture, the cells were in the log phase with a growth rate of 0.5 to 0.8/day. This was used for the experiment, which was meant mainly for two purposes (a) to assess whether there is any noticeable change in chlorophyll *a* concentration in individual phytoplankton cell when large changes occur in light conditions, and (b) to understand through pigment analysis whether there is any photoprotective or photo-adaptive mechanism present in the phytoplankton during large variations in PAR. The incubation experiments were conducted using all the three dominant phytoplankton species under different light intensities. All experimental bottles were incubated in triplicates, and neutral density filters were used for light cut-off to simulate the low light intensities. The different light levels inside the experimental bottles were created by adjusting the number of layers of the neutral density filters over the bottles. The PAR inside the experimental bottles was measured using a handheld PAR sensor (Apogee Quantum Flux, MQ- 200). With respect to the natural PAR available during the experimental period, 3 different PAR intensities were maintained in the experimental bottles − 100% (high), 10% (low) and 1% (lowest). The incubation of the experimental bottles was carried out for 4 hours during the peak daylight hours (10 to 14 hours). In accordance with the daily cycle, PAR over the incubation period also fluctuated from 400–1500 µmol m^−2^ s^−1^, and therefore, in the present case, ‘high’ condition encompasses PAR variation from 400–1200 µmol m^−2^ s^−1^, ‘low’ condition from 40–120 µmol m^−2^ s^−1^ and the ‘lowest’ condition from 4–12 µmol m^−2^ s^−1^. ‘Control’ in this experimental set up was intended to simulate the PAR (150–300 µmol m^−2^ s^−1^) observed in the field during the experimental sample collection. The same PAR range was also maintained in the environmental chamber to develop the cultures for the experiments.

Chlorophyll *a* per cell, light harvesting pigment (LHP), and photo protective pigments (PPP) in the dominant diatoms under different light conditions were measured following the experimental procedures given below. Firstly, the experimental phytoplankton suspension was prepared by dispensing 200 ml of the three diatom monocultures into separate transparent Nalgene bottles containing 20 liter of G/F filtered seawater from the North-western Bay of Bengal. All three diatom suspensions (1.5 liter each) were filled into triplicates of 2liter Borosil experimental bottles to have one set of ‘control,’ and three sets of ‘experimental’ bottles (representing light intensities high, low and lowest. All the experimental bottles were positioned at 0.5 m in the water column in a large FRB tank and incubated under natural light for 4 hrs. The FRB tank was equipped with continuous water overflow facility to ensure that the temperature was maintained close to the *in-situ* condition. The surface and 0.5 m PAR values in the FRB were measured every half an hour using the handheld PAR sensor (Apogee Quantum Flux, MQ- 200). After the incubation of the experimental bottles, from each set of the bottles, two 500 ml water samples were filtered through G/F filter papers, one sample for chlorophyll *a* measurements using a Turner fluorometer as per standard methodology^36^ and the other for analysing the photosynthetic pigments using HPLC^45^. Another 300 ml of water sample from each experimental bottle was analysed for phytoplankton cell counts based on a FlowCAM in the case of *Skeletonema* and an inverted microscope for *Chaetoceros* and *Thalassiosira*. Samples of chlorophyll *a*, phytoplankton counts and HPLC pigments were collected at the start and end of the incubation experiments. Chlorophyll *a* concentration per cell of phytoplankton was calculated by dividing the total chlorophyll a concentration by the total phytoplankton cell counts.

### Data availability

The datasets will be published in the public data repository of CSIR-National Institute of Oceanography (CSIR-NIO). It can be accessed by anybody after submission of individual request to Director CSIR-NIO or Scientist-in-charge of CSIR-NIO Regional Centre, Kochi, India as per the Institutional policy.

## Electronic supplementary material

Supplementary Information 1
