# Supplementary material for: Response of phytoplankton to heavy cloud cover and turbidity in the northern Bay of Bengal
Source: Sci Rep. 2018 Jul 26;8:11282. doi: 10.1038/s41598-018-29586-1 (PMC6062583; doi:10.1038/s41598-018-29586-1)
Supplement: Supplementary file 1 — Supplementary Information 1 [file 41598_2018_29586_MOESM1_ESM.docx]

Title:

‘Response of phytoplankton to heavy cloud cover and turbidity in the northern Bay of Bengal’

Authors:

R. Jyothibabu, Arunpandi, N., Jagadeesan, L., Karnan, C., Lallu, K.R., P.N. Vinayachandran

**Supplementary Information 1** - Regression between CTD sensor and lab flourometer chlorophyll *a* data
